# Efficient implied alignment

**DOI:** 10.1186/s12859-020-03595-2

**Published:** 2020-07-09

**Authors:** Alex J. Washburn, Ward C. Wheeler

**Affiliations:** Division of Invertebrate Zoology, American Museum of Natural History, 200 Central Park West, New York, 10024-5192 NY USA

**Keywords:** Dynamic homology, Implied alignment, Multiple string alignment, Phylogenetics, Sequence alignment, Tree alignment

## Abstract

**Background:**

Given a binary tree $\mathcal {T}$ of *n* leaves, each leaf labeled by a string of length at most *k*, and a binary string alignment function ⊗, an implied alignment can be generated to describe the alignment of a dynamic homology for $\mathcal {T}$. This is done by first decorating each node of $\mathcal {T}$ with an alignment context using ⊗, in a post-order traversal, then, during a subsequent pre-order traversal, inferring on which edges insertion and deletion events occurred using those internal node decorations.

**Results:**

Previous descriptions of the implied alignment algorithm suggest a technique of “back-propagation” with time complexity $\mathcal {O}\left (k^{2} * n^{2}\right)$. Here we describe an implied alignment algorithm with complexity $\mathcal {O}\left (k * n^{2}\right)$. For well-behaved data, such as molecular sequences, the runtime approaches the best-case complexity of *Ω*(*k*∗*n*).

**Conclusions:**

The reduction in the time complexity of the algorithm dramatically improves both its utility in generating multiple sequence alignments and its heuristic utility.

## Background

Implied Alignment (IA) was proposed by [1] as an adjunct to Direct Optimization (DO) [2, 3] to be used in phylogenetic tree search to provide both verification and more rapid heuristic analysis. The method was originally implemented in later versions of MALIGN [4] and has been a component of POY [5–9] since its inception. A more formal description of the algorithm was presented in [6] and [2]. Although originally designed for alignment-free phylogenetic analysis (dynamic homology, [10]), the procedure was first used as a stand-alone multiple sequence alignment (MSA) tool by [11] in their analysis of skink systematics.

IA was originally described in the context of parsimony-based phylogenetic analysis and was later extended to probabilistic model-based approaches by [12] and its implementations were described by [9, 13]. Similar MSA approaches also based in probabilistic analysis have been described e.g by [14] and [15], and implemented in PRANK [16]. Whiting et al. found that IA was superior (in terms of tree optimality score) to other MSA methods in both parsimony and likelihood analyses. This observation has been repeated multiple times (e.g. [17–20]; summarized in [21]). The use of IA as an MSA algorithm as well as its use in the “static approximation” procedure [22] benefits greatly from improvements in the time complexity we present in this paper.

In a broader context, IA is a heuristic solution to the NP-hard Tree Alignment Problem (TAP) defined by [23]. As such, any individual IA is not guaranteed to be either optimal or unique, with potentially an exponential number of equally optimal implied alignments for any given binary tree.

The IA algorithm presented in this paper takes a different intellectual approach to deriving alignments than earlier versions of IA. Previous algorithmic approaches relied on DO assigning median sequences to the graph vertices. These sequences were then consumed by IA to produce the full alignment. Here, we describe IA as assigning “preliminary contexts” to the graph vertices, and later consuming these *contexts* to produce the median sequences and the full alignment.

The algorithm uses the repeated application of a pairwise string alignment function to perform an efficient MSA for a given binary tree whose leaves are labeled by strings, i.e. the tree describes the relationship of those strings. The more similar the initial leaf labelings the better the algorithm performs. Thus, while this algorithm has general use for performing an MSA, it is especially well-suited for the alignment of biological sequences where the strings are highly similar and a binary tree describing the strings’ relationships can be provided. Below, we provide an example of the IA algorithm’s performance on biological data.

## Definition of the heuristic function

In order for an MSA to be inferred, there are constraints on the heuristic alignment function used to decorate the tree prior to performing the IA algorithm. As long as these constraints are satisfied, the implementation details of the function are agnostic to the IA algorithm presented here.

Let *Σ* be a finite alphabet of symbols such that |*Σ*|≥3. Let (−)∈*Σ* be a gap symbol, which will have a special meaning in the context of an alignment. Let $\mathcal {P}_{\geq 1}({X})$ denote the powerset of X, minus the empty set. Let *Σ*_*Γ*_ be the alphabet of the following symbols:

$$\begin{array}{*{20}l} \Sigma_{\Gamma} & = \text{\texttt{BOTH}} \;\;\;\; \mathcal{P}_{\geq1}(\Sigma) \;\; \mathcal{P}_{\geq1} (\Sigma) \\ & \;\, | \;\; \text{\texttt{LEFT}} \;\;\;\; \mathcal{P}_{\geq 1} (\Sigma) \\ & \;\, | \;\; \text{\texttt{RIGHT}} \;\; \quad\quad\quad\, \;\; \mathcal{P}_{\geq 1} (\Sigma) \\ & \;\, | \;\; \text{\texttt{GAPPED}} \end{array} $$

That is, *Σ*_*Γ*_ contains all pairs of elements of $\mathcal {P}_{\geq 1} (\Sigma)$ tagged as BOTH, all elements of $\mathcal {P}_{\geq 1} (\Sigma)$ tagged as LEFT, all elements of $\mathcal {P}_{\geq 1} (\Sigma)$ tagged as RIGHT, and an additional element GAPPED. This construction of *Σ*_*Γ*_ extends the original alphabet *Σ* to preserve alignment information in the algorithms presented below. Note that if |*Σ*|=*x* then |*Σ*_*Γ*_|=2^2*x*^. This follows from the fact that $|\mathcal {P}_{\geq 1} (\Sigma)|$ is equal to one less than the size of the power set of *Σ*, due to $\mathcal {P}_{\geq 1} (\Sigma)$ disallowing the empty set.

Let $\Sigma ^{*}_{\Gamma }$ be the set of all finite strings over the alphabet *Σ*_*Γ*_. Let $\otimes : \Sigma ^{*}_{\Gamma } \times \Sigma ^{*}_{\Gamma } \rightarrow \left (\mathbb {R}_{\geq 0},\; \Sigma ^{*}_{\Gamma }\right)$ be a heuristic function that returns a nonnegative alignment cost and an alignment result in $\Sigma ^{*}_{\Gamma }$. It is required that ⊗ be commutative but it need not be associative. Both of these constraints will be explored later in the “Discussion” Section. These constraints are necessary but not sufficient for a heuristically optimal implied alignment to be inferred on the alignment function.

It is worth noting the motivation of the constructions defined above. Most pairwise string alignment functions take two finite strings of symbols from the original alphabet and supply a new finite string of symbols from the original alphabet. We can represent this class of pairwise string alignment functions by letting *Σ*^∗^ be the set of all finite strings over the alphabet $\mathcal {P}_{\geq 1} (\Sigma)$ and letting $\odot : \Sigma ^{*} \times \Sigma ^{*} \rightarrow \left (\mathbb {R}_{\geq 0},\; \Sigma ^{*} \right)$. The results of ⊙ can contain cases of ambiguity where it cannot be inferred which input elements correspond to which output elements, but the construction of ⊗ never produces these cases of ambiguity due to the tagging of each element. The preservation of this non-ambiguous relationship between input and output is required for the algorithmic improvements presented below.

## Overview of the implied alignment algorithm

The IA algorithm provides an MSA for a binary tree $\mathcal {T}$ of *n* leaves, each leaf containing a string with symbols in *Σ* and length at most *k*. To generate this alignment, we will traverse $\mathcal {T}$ twice. First, we perform a post-order traversal—from the leaves to the root—assigning the results of ⊗ as a “preliminary context” decoration to each node. Second, we perform a pre-order traversal—from the root to the leaves—aligning each preliminary context with its parent to assign a “final alignment” decoration to each node.

Using a binary string alignment function (like ⊗) to produce an MSA efficiently relies on the output of binary operations combined across the “global scope” of $\mathcal {T}$. However, at each step in the post-order traversal, the only information known at a given node is the information contained in its subtree. Therefore, information for the entirety of $\mathcal {T}$ is only known at completion of the post-order traversal. When performing the subsequent pre-order traversal, we take the “complete” scope available at the root node and thread the information towards the leaves. At each pre-order step, we take the “complete” context threaded from the root and combine it with the preliminary context derived during the post-order pass to assign the final alignment on that node. Thus, we collect all requisite information for an MSA during the post-order traversal and then apply that information during the pre-order traversal to derive the MSA.

As noted above, the time complexity of the IA algorithm’s pre-order traversal in previous work was $\mathcal {O}\left (k^{2} * n^{2}\right)$. We are able to improve this by, during the post-order pass, tagging each element of a string on a node *v*_*x*_ with information that notates on which subtree of *v*_*x*_ that element originated. We can lift each symbol in *Σ* into *Σ*_*Γ*_ through the alignment process. Upon completion of the post-order traversal, each node in $\mathcal {T}$ will have a string in $\Sigma _{\Gamma }^{*}$. Each element of said strings are tagged with one of four options enumerated above, representing from which child node the information of that element originated, relative to that node. Those tagged BOTH originated from both subtrees, those tagged LEFT originated from the left subtree, those tagged RIGHT originated from the right subtree, and those tagged GAPPED originated from neither subtree (i.e., elsewhere in $\mathcal {T}$). Because elements tagged GAPPED originated from neither subtree, GAPPED those elements cannot be created during the post-order, only derived during the pre-order.

This tag on each element provides contextual information that allows for an efficient processing of the elements in the pre-order traversal. During the pre-order traversal, a node’s preliminary context is “zipped” with the parent’s alignment in order to derive its final alignment. We will show that this tagging and “zipping” process is a substantial improvement over previous work, reducing the time complexity from quadratic to linear in the length of the strings. It is worth noting that this tagging can be represented as a succinct data structure per [24], requiring only two additional bits per element.

## An example heuristic function

We will provide an example definition of ⊗ in Algorithm 1 sufficient for the IA algorithm, though there are other sufficient definitions of ⊗. The candidate function fitting the description of ⊗ we present will be defined as a extension of the Needleman-Wunsch [25] algorithm for pairwise string alignment. The algorithm is modified along the same the lines that DO modified the dynamic programming technique of [26], with an additional step taken to produce the tagged elements in the output alignment. Algorithm 1 (described below) is used to generate the results presented in the “Methods” section.


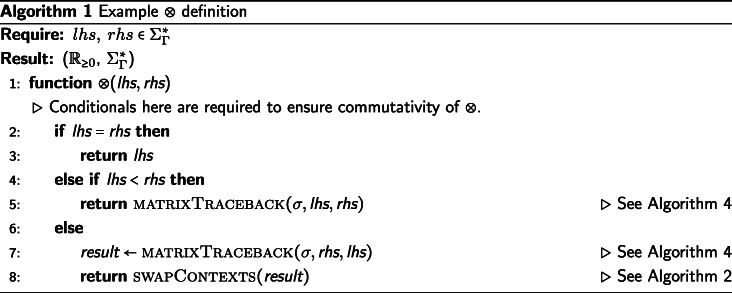



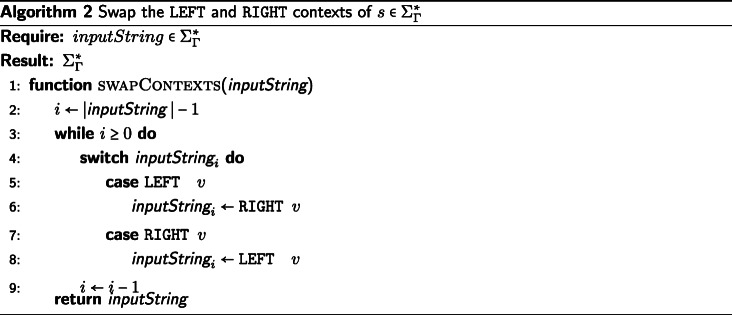


First, we decide deterministically which of the two input strings is assigned to the top (columns) of the alignment matrix and which string is assigned to the left side (rows). We assign the input strings based on the data they contain. The longer string is assigned to the columns of the alignment matrix, the shorter string to the rows. If the strings are the same length, we take the first string under the lexical ordering of their elements and assign it to the columns and assign the second string to the rows of the alignment matrix. In the case that the strings are identical, the alignment is trivial. If the first string supplied to ⊗ was not assigned to the rows of the matrix, then we must swap the LEFT and RIGHT tags of the resulting string alignment before returning the result. This consistency in assignment ensures the commutativity of ⊗, which is necessary to enforce consistency of the IA algorithm. Commutativity of ⊗ ensures that the IA algorithm provides the same alignment results for isomorphic tree labeling (i.e. ensures label invariance).

We now apply a memoized update procedure [27], a common element of dynamic programming algorithms such as the Needleman-Wunsch alignment. The subsequent “traceback,” however, is notably modified from the original Needleman-Wunsch procedure. The upward, leftward, and diagonal directional arrows used to produce the alignment are additionally used to tag each element as LEFT, RIGHT, or BOTH, respectively. These tagged pairwise alignments will be consumed on the subsequent pre-order traversal of $\mathcal {T}$ when merging preliminary contexts. Storing this information for each element of the pairwise alignment allows a more efficient generation of the subsequent multiple string alignment, allowing for an asymptotic improvement over the previous IA algorithm. This additional tagging detail is the key difference between previous alignment methods and the one presented in this paper.

The example ⊗ presented in Algorithm 1 is of *Θ*(*k*^2^) complexity in both time and space, where *k* is the length of the longer string. For clarity, while this example function is presented as a modification of the well understood Needleman-Wunsch algorithm (without explicit memoization), this tagging approach can be incorporated into more sophisticated pairwise string alignment algorithms. For instance, by using the method described by [28], this algorithm’s time complexity could be improved to $\mathcal {O}\left (k * s \right)$, where *s* is the edit distance between the strings. Alternatively, by using the method described by [29], this algorithm could be improved to use $\mathcal {O}\left (k \right)$ space. Affine gap models [30] can also be incorporated via the method of [2].

The operator $\sigma \,:\, \mathcal {P}_{\geq 1}(\Sigma) \times \mathcal {P}_{\geq 1}(\Sigma) \rightarrow \left (\mathbb {R}_{\geq 0},\, \mathcal {P}_{\geq 1}(\Sigma) \right)$ presented in Algorithms 1, 3, and 4 represents a metric for determining the transition cost between symbols in $\mathcal {P}_{\geq 1} (\Sigma)$. The metrics used in our data sets can be found in Table 1. The metrics presented in Table 1 show the transition cost between elements of *Σ*. However, these metrics can be expanded to define the transition costs between elements of $\mathcal {P}_{\geq 1}(\Sigma)$ in the manner described by [2]. Note that *σ* can also be a more complex metric than those presented here, for instance a metric with affine or logarithmic affine gap costs, and be compatible with the IA algorithm. For usage of *σ*, see Algorithms 1, 3, and 4.

**Table 1:** Metric costs of *σ*_0_, *σ*_1_, and *σ*_2_

*σ*_0_	A	C	G	T	–		*σ*_1_	A	C	G	T	–		*σ*_3_	A	C	G	T	–
A	0	1	1	1	2		A	0	3	3	3	1		A	0	1	1	1	1
C	1	0	1	1	2		C	3	0	3	3	1		C	1	0	1	1	1
G	1	1	0	1	2		G	3	3	0	3	1		G	1	1	0	1	1
T	1	1	1	0	2		T	3	3	3	0	1		T	1	1	1	0	1
–	2	2	2	2	0		–	1	1	1	1	0		–	1	1	1	1	0

Expansion of the metrics presented in Table 1 is described by [2]


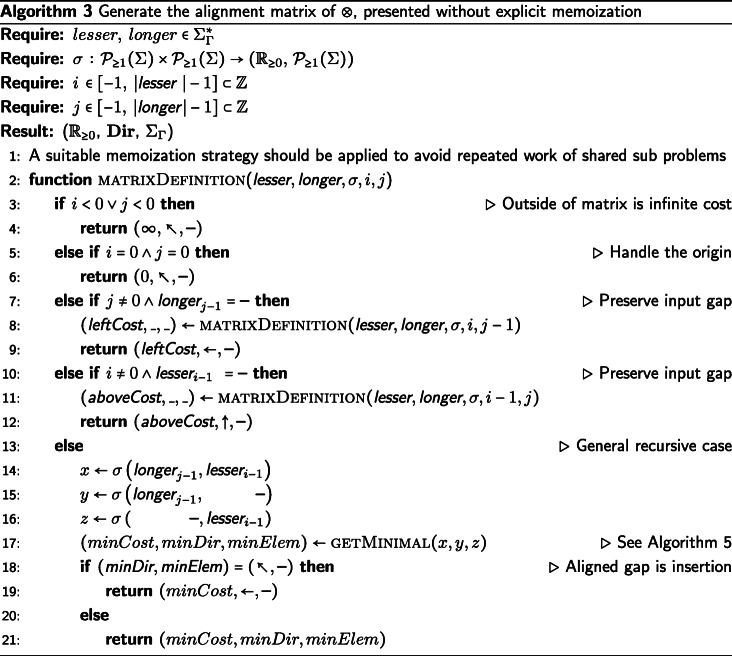


## Description of post-order traversal

The post-order traversal (leaves to the root) of the binary tree $\mathcal {T}$ is a straightforward procedure, see Algorithm 6. We assign preliminary contexts and costs to each node, *v*_*x*_, of $\mathcal {T}$. These preliminary contexts will be consumed to assign a final alignment in the subsequent pre-order traversal of the tree. The post-order traversal described here is very similar to the DO post-order traversal described by [1], differing only in the use of ⊗ which captures the preliminary context of a subtree, instead of generating a preliminary median string assignment.

First, for each leaf node, *v*_*x*_, we set *v*_*x*_.*cost* to 0. Additionally, if *v*_*x*_ is of type *Σ*^∗^ and not of type $\Sigma ^{*}_{\Gamma }$—i.e. if it has been decorated with a finite string of symbols from the alphabet *Σ*, and it is not decorated with a finite string of preliminary contexts over the alphabet *Σ*_*Γ*_—then we call INITIALIZESTRING(*v*_*x*_.*prelimString*) to apply the transformation $\Sigma ^{*} \rightarrow \Sigma ^{*}_{\Gamma }$.

On each internal node, *v*_*y*_ with children *v*_*l*_ and *v*_*r*_, of $\mathcal {T}$, we call *v*_*l*_ ⊗ *v*_*r*_. The resultant *prelimString* is assigned to *v*_*y*_.*prelimString*, and the sum of the *v*_*l*_.*cost*, *v*_*r*_.*cost*, and the alignment cost of *v*_*l*_ ⊗ *v*_*r*_ is assigned to *v*_*y*_.*cost*. By performing this operation in a post-order traversal over $\mathcal {T}$, we propagate the preliminary contexts and costs returned from the calls to ⊗ from the leaves to the root.


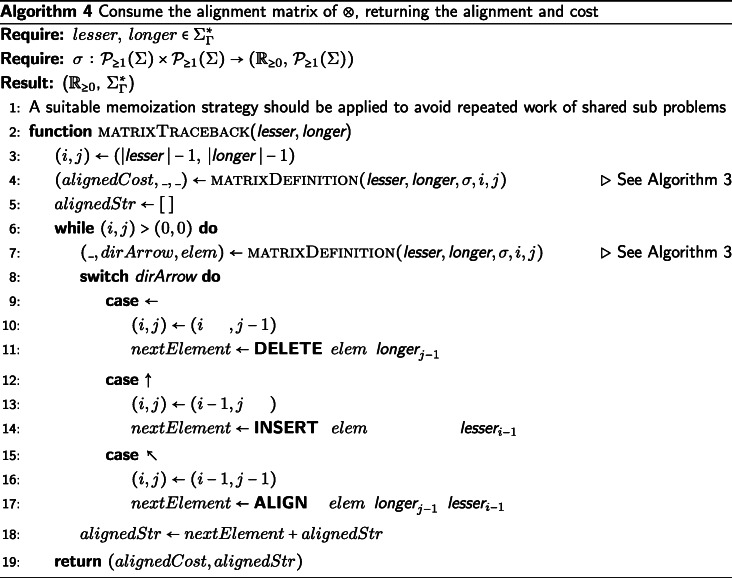


Upon completion of the post-order traversal, each internal node contains the preliminary context information and the cost for the corresponding subtree. Consequently, when the post-order traversal is complete, the root node contains the preliminary context information of the full leaf set of strings and the alignment cost for the entire tree $\mathcal {T}$. In the pre-order traversal, we will consume this preliminary context to perform an (efficient) alignment on the strings.

Because the post-order traversal can be performed using any valid definition of ⊗, the complexity of the post-order traversal is dependent on the complexity of the heuristic alignment function used. Let the complexity of ⊗ be defined as *H*(*k*), where *k* is the maximum string length of the leaf labels of the tree $\mathcal {T}$. Then post-order traversal runs in $\mathcal {O}(H(k) * n)$ time and space, where *n* is the number of leaves in the binary tree $\mathcal {T}$. If we were to use Ukkonen’s method with the ⊗ described in Algorithm 1, the post-order traversal would run in $\mathcal {O}(k * s * n)$ time and space, where *s* is the maximum edit distance between any two strings.

## Description of pre-order traversal for final alignments

The pre-order traversal (from the root to the leaves) of the binary tree $\mathcal {T}$ consumes the preliminary context decorations on each node created in the post-order traversal in order to assign final alignment decorations of $\Sigma _{\Gamma }^{*}$ to each node, see Algorithm 7. First, the root node must be initialized for the pre-order traversal by assigning the root’s preliminary context to the root’s final alignment. By initializing the root node in this manner, the root node is consistent with the treatment of any other parent node when deriving the internal node alignments in Algorithm 8.


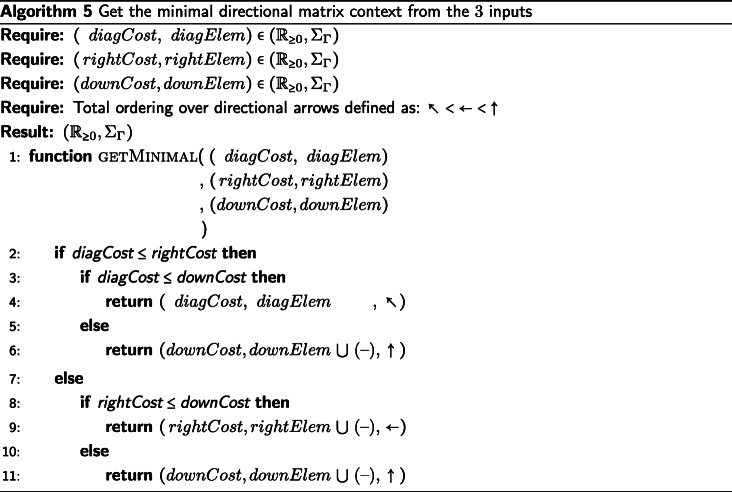



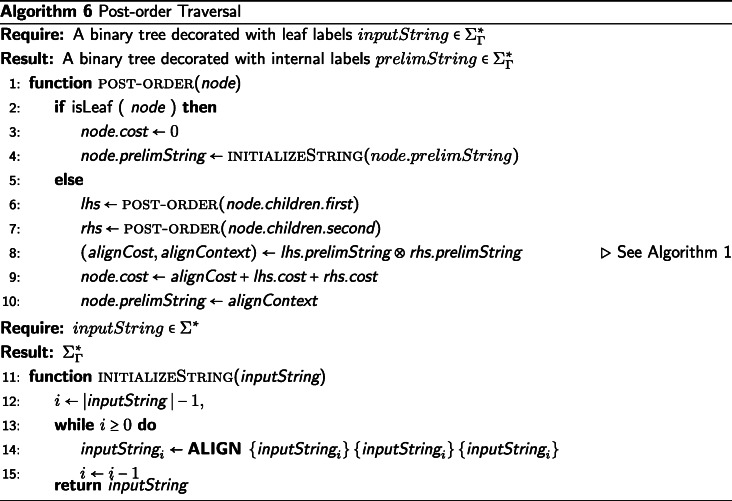



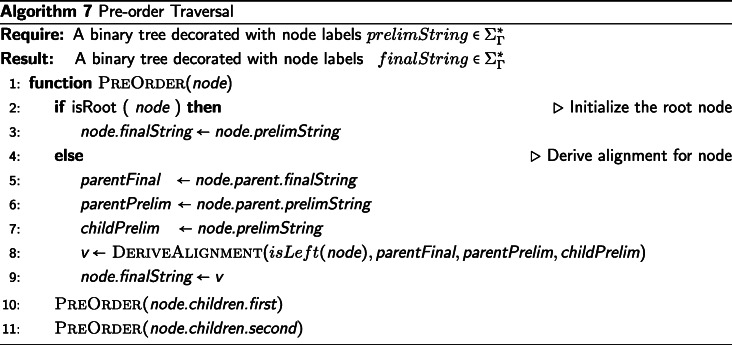



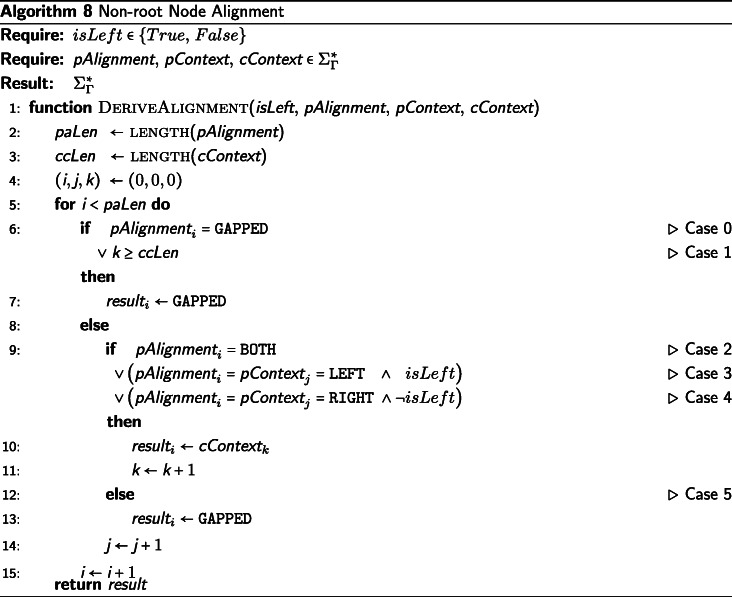


For each non-root node, *v*_*c*_, we first determine whether *v*_*c*_ is the left or right child of its parent. This is required because LEFT-tagged elements originate from alignments of the left subtree and RIGHT-tagged elements originate from alignments of the right subtree, and we must use this information when deriving the final alignment of *v*_*c*_.

The final alignment of the parent of *v*_*c*_, *v*_*p*_, will necessarily be of greater than or equal length to *v*_*p*_’s preliminary context, because *v*_*p*_’s final alignment contains all the information from the contexts of *v*_*p*_’s subtrees as well as the information from the rest of the tree, that is, the contexts of all of the subtrees of every ancestor node to *v*_*p*_. The preliminary context of *v*_*p*_ is also of greater than or equal length to preliminary context of *v*_*c*_, due to the *v*_*p*_’s context containing all information from *v*_*c*_’s context, plus the addition of *v*_*c*_’s sister subtree. The resulting value assigned to *v*_*c*_’s final alignment will have the same length as the final alignment assigned to *v*_*p*_. Since this invariant length is maintained from the root node to the leaf nodes’ final alignment assignments, all alignments will have the same length. This constitutes a simple inductive argument that the final alignment assignment of each node will be of equal length and constitute a genuine string alignment.

**Fig. 1:**
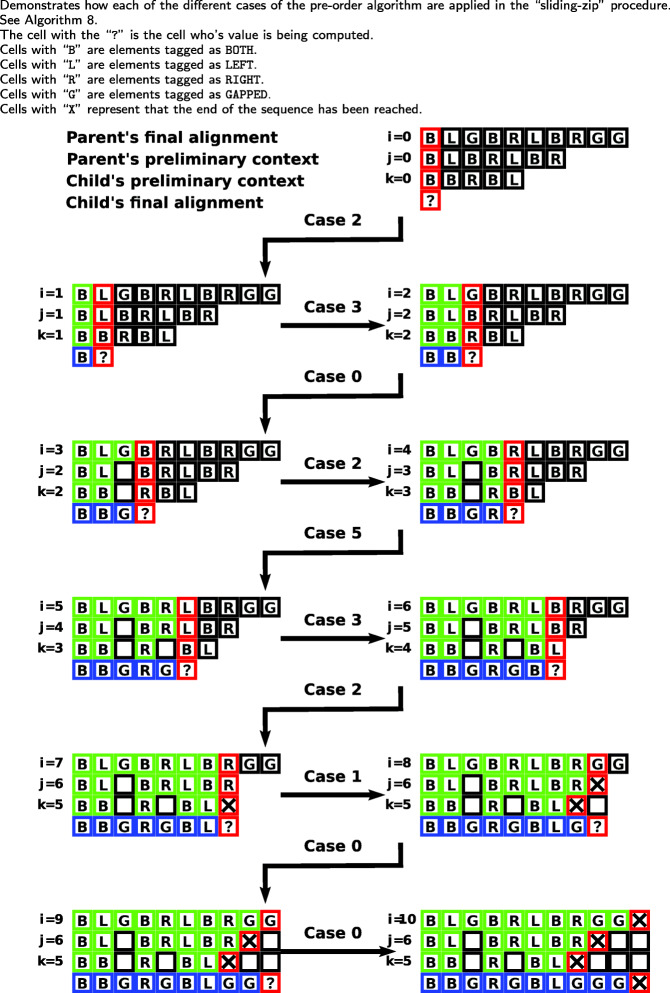
Example pre-order alignment for a parent node and it’s left child

The final alignment for *v*_*c*_ is derived by performing a “sliding zip” over *v*_*p*_’s final alignment, *v*_*p*_’s preliminary context, and *v*_*c*_’s preliminary context. *v*_*p*_’s final alignment is used as the basis of the zip. The inputs to this alignment are the preliminary contexts of *v*_*p*_ and *v*_*c*_ and the final alignment of *v*_*p*_. At each step of the “sliding zip,” one element of *v*_*p*_’s final alignment will be consumed and one element of *v*_*c*_’s final alignment will be defined. Additionally, at each step of the zip, one of: an element from *v*_*p*_’s preliminary context, elements from both *v*_*p*_’s and *v*_*c*_’s preliminary contexts, or no elements from either node’s context, will be consumed. Finally, we define an element of *v*_*c*_’s final alignment to be either an element from *v*_*c*_’s preliminary context or a gap. The process is called a “sliding zip” because, due to the varying lengths of the three inputs, the elements of *v*_*p*_’s and *v*_*c*_’s preliminary contexts do not have an immediately apparent index with which they correspond to *v*_*p*_’s final alignment, which is used as the basis of the zip. Rather, the elements of *v*_*p*_’s and *v*_*c*_’s preliminary contexts “slide” through the zipping process, and their corresponding indices with *v*_*p*_’s final assignment is deduced dynamically. The logic applied in the “sliding zip” is to propagate gaps from the final alignment of *v*_*p*_, which contains the gaps of the entire tree above *v*_*c*_, down to *v*_*c*_, and when not dealing with a gap propagated from an ancestor node to *v*_*c*_, to align the non-gap elements or introduce a new gap to be propagated. The “sliding zip” process is often easier to understand by stepping through the algorithm. An example alignment of this “sliding zip” process for two internal nodes is shown in Fig. 1.

There are five cases determining the derivation of each index of *v*_*c*_’s final alignment. The cases are presented in the pseudocode of Algorithm 8, in Fig. 1, and described below.

- Case 0: When the element of *v*_*p*_’s final alignment is “GAPPED,” then the next element of *v*_*c*_’s final alignment is “GAPPED.”
- Case 1: When the sliding zip has consumed all elements of the *v*_*c*_’s preliminary context, then the next element of *v*_*c*_’s final alignment is “GAPPED.” Because we only define the next element of *v*_*c*_’s final alignment to be either an element from *v*_*c*_’s preliminary context or a gap, the latter is the only choice.
- Case 2: When the element of *v*_*p*_’s final alignment is “BOTH,” then we consume the next elements of both *v*_*p*_’s and *v*_*c*_’s preliminary contexts, and the next element of *v*_*c*_’s final alignment is *v*_*c*_’s consumed preliminary context element. Because *v*_*p*_’s final alignment element was marked as an alignment event, we know that *v*_*c*_ was aligned with its sister subtree at this index, and that *v*_*c*_’s preliminary context element is the correct element for this index of the alignment.
- Case 3: When both *v*_*p*_’s final alignment element and *v*_*p*_’s preliminary context element are “LEFT,” and *v*_*c*_ is the *left* child of *v*_*p*_, then we consume the next element of each of *v*_*p*_’s and *v*_*c*_’s preliminary contexts, and the next element of *v*_*c*_’s final alignment is *v*_*c*_’s consumed preliminary context element. Because LEFT-tagged elements originate from the left subtree of a node, and *v*_*c*_ is the left child of *v*_*p*_, *v*_*c*_’s preliminary context element is the correct element for this index of the alignment. If the same LEFT-tagged elements were encountered but *v*_*c*_ was the right child of *v*_*p*_, then *v*_*c*_’s preliminary context element would not be the correct element for this index of the alignment, because LEFT-tagged elements originate from the left subtree of *v*_*p*_ and the LEFT-tagged element under consideration was encountered in *v*_*p*_’s right subtree. In the case that a LEFT-tagged element is encountered in the right subtree of *v*_*p*_, we introduce a new gap into all the alignments of the subtree at this index to account for the aligned element in the *v*_*c*_’s sister subtree. This is implicitly dealt with in Case 5.
- Case 4: Conversely to Case 3, when both *v*_*p*_’s final alignment element and *v*_*p*_’s preliminary context element are “RIGHT,” and *v*_*c*_ is the *right* child of *v*_*p*_, we consume the next element of both *v*_*p*_’s and *v*_*c*_’s preliminary contexts and assign to the next element of *v*_*c*_’s final alignment *v*_*c*_’s consumed preliminary context element. Because RIGHT-tagged elements originate from the right subtree of a node, and *v*_*c*_ is the right child of *v*_*p*_, *v*_*c*_’s preliminary context element is the correct element for this index of the alignment. If the same RIGHT-tagged element was encountered but *v*_*c*_ was the left child of *v*_*p*_, then *v*_*c*_’s preliminary context element would not be the correct element for this index of the alignment, because RIGHT-tagged elements originate from the right subtree of *v*_*p*_ and the RIGHT-tagged element under consideration was encountered in *v*_*p*_’s left subtree. In the case that a RIGHT-tagged element is encountered in the left subtree of *v*_*p*_, we introduce a new gap into all the alignments of the subtree at this index to account for the aligned element in *v*_*c*_’s sister subtree. This is implicitly dealt with in Case 5.
- Case 5: When none of the conditions for Case 0, 1, 2, 3, or 4 hold, then we consume the next element of *v*_*p*_’s preliminary context and the next element of *v*_*c*_’s final alignment is “GAPPED.” This handles the cases where either the two subtrees were not aligned at the current index or a new gap needed to be introduced at the current index because a LEFT-tagged or RIGHT-tagged element was encountered in *v*_*p*_’s right or left subtree, respectively.

## Analysis of pre-order traversal

Let $m = \frac {a}{k}$, where *k* is the length of the longest input string, and *a* is the length of the root node’s preliminary context. In the best case that a “perfect alignment” is derived, that is, that each element of all the input strings can be aligned with one of the elements of the longest input string, then *m*=1. In the worst case that a “degenerate alignment” is derived, that is, that no element of any of the input strings can be aligned with any of the elements of the longest input string, and all input strings are of equal length, then *m*=*n*.

The improvement of the implied alignment algorithm presented here compared to the original algorithm is that the additional stored information allows us to determine the final assignments in *Θ*(*k*∗*m*∗*n*) instead of $\mathcal {O}\left (k^{2} * n^{2}\right)$ time. The aforementioned *n*^2^ component occurred in previous implementations due to the use of a “back-propagation” technique, which required that, at each pre-order step, each new gap found in the alignment was retroactively applied to every alignment derived in a previous pre-order step. The *k*^2^ component in the previous implementation was due to using a Needleman-Wunsch string alignment between the current node and its parent node at each pre-order step in addition to the alignment already performed at each post-order step. By saving the requisite information on the nodes during the post-order traversal and then consuming this information with the “sliding-zip” technique, we eliminate the Needleman-Wunsch alignments during the pre-order, as well as the back-propogation, and replace these computationally expensive operations with a much more efficient algorithm.

In the pre-order traversal algorithm presented above, we generate an implied alignment in *Θ*(*k*∗*m*∗*n*) time. We must perform a “sliding-zip” operation on each node in the binary tree $\mathcal {T}$, hence the factor of *n*. The “sliding zip” accounts for the *k*∗*m* factor.

The best case time complexity occurs when the length of the derived alignment is the length of the longest input string, an alignment with the minimal number of elements. In this case, *m*=1 and the “sliding zip” performed on each node performs work equal to the length of the longest input string *k*. Hence, the best case time complexity of the implied alignment algorithm is *Ω*(*n*∗*k*), occurring when the input strings are highly correlated and *m*=1.

The worst case time complexity occurs when the length of the derived alignment is equal to the sum of the lengths of the input strings, an alignment with the maximum number of elements. In the worst case, *m*≫1, and the “sliding zip” performed on each node performs work equal to the length of the longest input string, *k*, multiplied by the number of input strings, *n*. Hence, the worst case time complexity of the implied alignment algorithm is $\mathcal {O}(k * n^{2})$, occurring when the input strings are independent of each other.

## Methods

An example Haskell implementation of the implied alignment algorithm described above, the data sets used to generate the results, along with a script to replicate the results discussed below can all be found here: https://github.com/recursion-ninja/efficientimplied-alignment/replicate-results.sh

We ran the implied alignment algorithm described in this paper on a pathological data set that was constructed to illustrate the best and worst case performances of the implied alignment algorithm. The pathological data set consisted of balanced binary trees which repeatedly doubled in size. The smallest tree is a quartet tree, with the strings consisting of a single symbol from the alphabet *Σ*={A,C,G,T} repeated *k* number of times. The lengths of the strings on each leaf were repeatedly doubled in size to scale the string length. The size of the tree was scaled by taking $2^{\frac {n}{4}}$ quartet trees and combining them together into a larger balannced binary tree of *n* leaves.

The time complexity scaling of this pathological data set was examined under two different metrics, *σ*_0_ and *σ*_1_. The former metric preferentially selects substitution events over insertion or deletion elements, thus producing the “perfect alignment.” Conversely, the latter selects for insertion or deletion over substitution, thus producing the “degenerate alignment.”

Additionally, to explore the performance of the pre-order traversal on “real world” data, the algorithm was run on the fungal and metazoan biological data sets described by [31] and [32] respectively. Both full data sets consisted of a preselected tree and predetermined string alignment (i.e. including gaps). The full leaf set of the tree was repeatedly halved to produce a data set of doubling leaf set sizes. The string alignment was repeatedly truncated, dropping the beginning and end of the alignment, taking the central slice of the current length from each string, and then removing all the gaps from the alignment slice. The pruned trees and truncated strings were used as progressively doubling inputs, to measure runtime scaling in terms of both leaf set size and string length. Both biological data sets used the discrete metric *σ*_3_ and the alphabet *Σ*={A,C,G,T, – }.

After running the algorithm on each data set, we constructed an Ordinary Least Square (OLS) model with the running time in milliseconds as a function of dimensions *k* and *n*. We took the binary logarithm, log2, of both input dimensions as well as the output. From there, we calculated the coefficients of each input in this equation: log2(*runtime*)=*β*_0_+*β*_1_ log2(*n*)+*β*_2_ log2(*k*)+*ε*, where *ε* represents the estimation error. Note that, because the logarithm of the inputs was taken, we would expect *β*_1_ to be close to 1 for linear performance with respect to that input variable and close to 2 for quadratic performance. See Table 2.

**Table 2:** Regression coefficients of leaf-set size and string length on runtime

	*Dependent variable:*			
	log2 (Runtime)			
	Best	Worst	Fungi	Metazoa
	(1)	(2)	(3)	(4)
log2 (String count *n*)	1.057	2.021	1.236	1.511
log2 (String length *k*)	0.920	1.120	0.836	1.038
Observations	49	49	42	42
Adjusted R^2^	0.990	0.996	0.987	0.995

A direct runtime comparison between the $\mathcal {O}\left (k^{2} * n^{2}\right)$ algorithm in POY and our improved algorithm was not readily achievable due to being implemented in different impure and purely functional languages, which come with confounding architectural designs. Instead we present the empirical runtime analysis of the pre-order traversal above. We did not thoroughly explore the implemented post-order traversal, as it does not deviate substantially from the well-understood Needleman-Wunsh algorithm. We have provided the reader a convenient https://github.com/recursion-ninja/efficient-implied-alignment/replicate-results.sh script in the aforementioned code repository to conduct their own analysis of both the pre-order and post-order traversals.

## Results

**Fig. 2:**
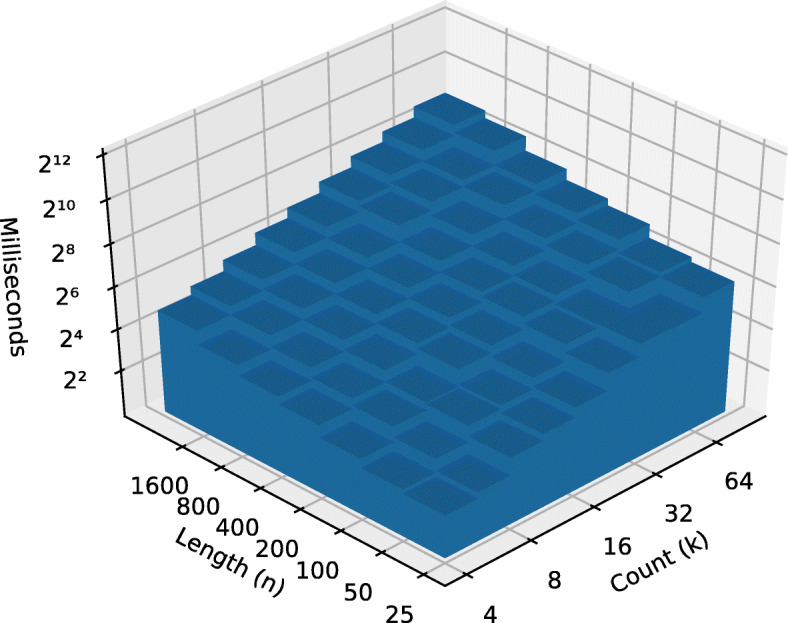
Best case pre-order runtime

**Fig. 3:**
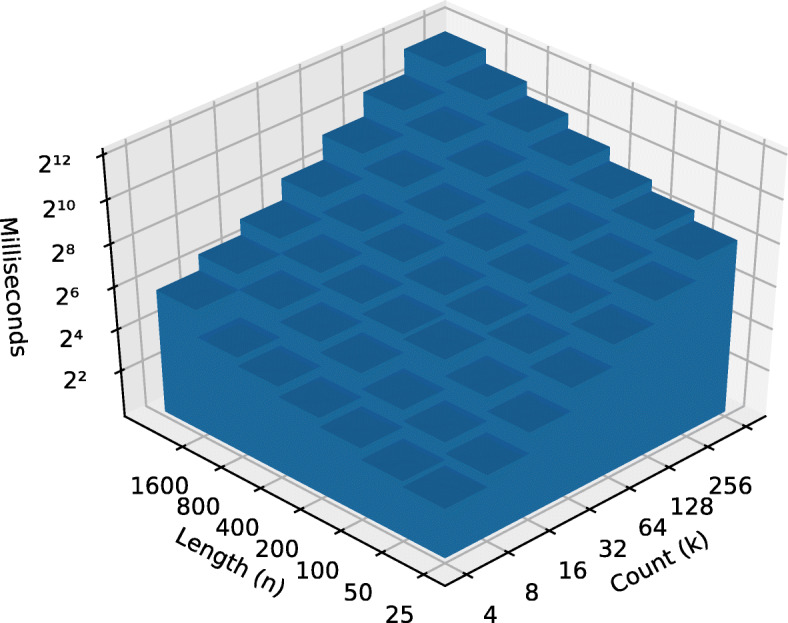
Worst case pre-order runtime

The pathological data sets shows the stark difference between the best case *Ω*(*n*∗*k*) and worst case $\mathcal {O}\left (k * n^{2}\right)$ performances. The OLS model empirically supports the theoretical best and worst cases demonstrated by the two runs on the pathological data set as shown in Figs. 2 and 3.

**Fig. 4:**
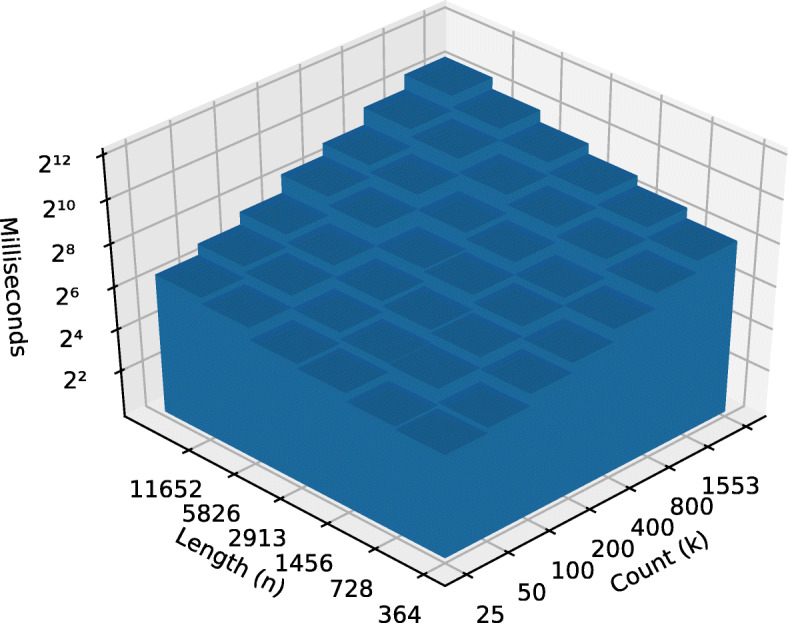
Fungi pre-order runtime

**Fig. 5:**
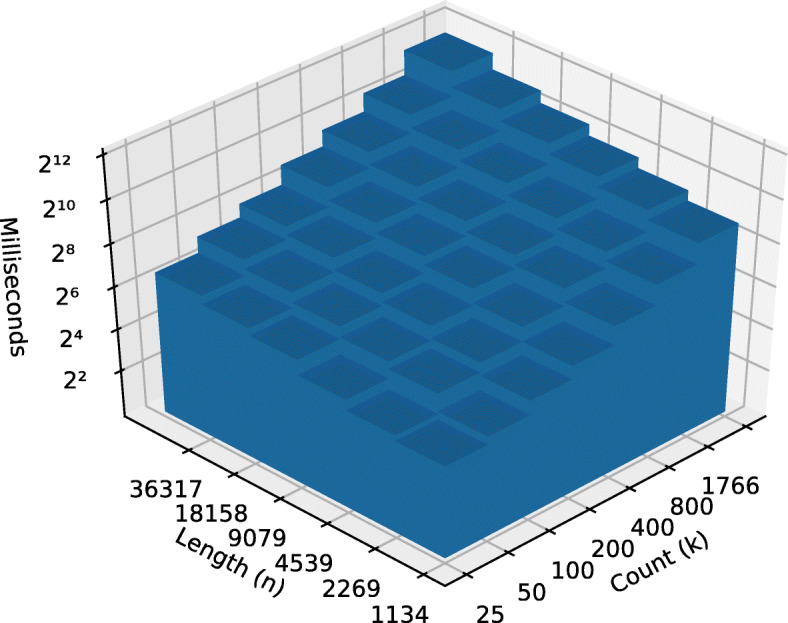
Metazoa pre-order runtime

The OLS model also anecdotally supports the supposition that time complexity scales well for the biological data sets. The fungal and metazoan sequence data sets demonstrate a near-linear time complexities with respect to the number of input strings and linear complexity with respect to string length. The fungal data sets lend support to the argument that some of “real world” use cases can perform close to the theoretical best case complexity (see Figs. 4 and 5).

## Conclusions

The IA algorithm can be improved to run with $\mathcal {O}\left (k * n^{2}\right)$ and best case *Ω*(*k*∗*n*) complexity of time and space. The more similar the input strings are, the closer the performance will be to the best case. When the algorithm is applied to “real world” biological sequences, the performance tends strongly towards the best case. The improved algorithm presented in this paper offers immediate and significant gains to applications related to the TAP and MSA.

## Discussion

The algorithm originally described by [1] was given the name implied alignment to differentiate it from other methods (e.g. sum-of-pairs alignment) unconnected to the vertex string assignments “implied” by the binary tree on a given leaf-set. However, it is worth articulating exactly how the alignment we derive is *implied* by the tree. In short, it is the requirement of commutativity and the lack of associativity.

For the purposes of this analysis we will ignore the cost returned from the ⊗ and consider only the resulting alignment context. Therefore let $\oplus : \Sigma ^{*}_{\Gamma } \times \Sigma ^{*}_{\Gamma } \rightarrow \Sigma ^{*}_{\Gamma }$ be defined as ⊗, but ignoring the alignment cost of the result. If we are given a rooted binary tree $\mathcal {T} = ((A,B),(C,D))$ with leaves *A*,*B*,*C*,*D*∈*Σ*^∗^ then the ancestral state of the root node defined by the heuristic function ⊕ would be ((*A*⊕*B*)⊕(*C*⊕*D*)). In fact, the ancestral state of any internal node defined by ⊕ can be calculated by applying ⊕ recursively to the subtree of the internal node. The binary structure of the tree directly implies the precedence of each application of ⊕ in the final result. Since ⊕ need not be associative, the tree ((*A*,(*B*,*C*)),*D*) evaluated as ((*A*⊕(*B*⊕*C*))⊕*D*), is likely to yield different results. However, since ⊕ is commutative, transposing any child nodes between the left and right positions of their parent will result in a tree that yields the same internal values. For example consider a transposed tree $\mathcal {T'}$:

$$\begin{array}{*{20}l} eval(\mathcal{T'}) &= eval((D,C),(B,A)) \\ &= ((D \oplus C) \oplus (B \oplus A)) \\ &= ((C \oplus D) \oplus (B \oplus A)) \\ &= ((C \oplus D) \oplus (A \oplus B)) \\ &= ((A \oplus B) \oplus (C \oplus D)) \\ &= eval((A,B),(C,D)) \\ &= eval(\mathcal{T}) \end{array} $$

This commutative property and lack of an associative property precisely determines that the alignment is implied by the tree on the leaf-set under ⊕ and not the unique alignment on all trees for the leaf-set under ⊕. Clearly, a ⊕ that is both commutative *and* associative using the algorithm described in this paper would yield the same alignment on all trees for a given leaf-set.

## Data Availability

The datasets generated and analysed in the study are available in the GitHub.com repository, https://github.com/recursion-ninja/efficient-implied-alignment

## Abbreviations

DO: Direct Optimization
IA: Implied Alignment
MSA: Multiple Sequence Alignment
OLS: Ordinary Least Square
TAP: Tree Alignment Problem
